# When Age and Culture Interact in an Easy and Yet Cognitively Demanding Task: Older Adults, But Not Younger Adults, Showed the Expected Cultural Differences

**DOI:** 10.3389/fpsyg.2017.00457

**Published:** 2017-03-27

**Authors:** Jinkyung Na, Chih-Mao Huang, Denise C. Park

**Affiliations:** ^1^Department of Psychology Sogang UniversitySeoul, South Korea; ^2^Department of Biological Science and Technology National Chiao Tung UniversityHsinchu, Taiwan; ^3^Center for Vital Longevity, University of Texas at Dallas, RichardsonTX, USA

**Keywords:** aged, culture, cognitive style, categorization, cultural differences

## Abstract

The interaction between age and culture can have various implications for cognition as age represents the effect of biological processes whereas culture represents the effect of sustaining experiences. Nevertheless, their interaction has rarely been examined. Thus, based on the fact that Asians are more intuitive in reasoning than Americans, we examined how this cultural difference might interact with age. Young and old participants from the US and Singapore performed a categorization task (living vs. non-living). To measure their reliance on intuition, we manipulated the typicality of targets (animate vs. inanimate). We showed that (1) RTs for inanimate organisms were slower than RTs for animate organisms (atypicality cost), (2) the cost was particularly large for older adults and (3) an age × culture interaction was observed such that cultural differences in the cost (Singaporeans > Americans) was found only among older participants. Further, we demonstrated that the age effect was associated with cognitive function and the culture effect among older adults was associated with cultural values. Finally, a moderated mediation analysis suggests that cognitive function and cultural values interact with each other in order to jointly influence one’s cognition.

## When Age and Culture Interact in Cognition: A Case of Categorization

As early as James (1890/1950), psychologists have investigated individual variations in cognition. Two important sources of differences in cognition are age and culture, each of which has been closely investigated. With increased age, adults show decreased performance in many cognitive domains, including processing speed, working memory, long-term memory, and reasoning; although measures of general knowledge are shown to be age-invariant (Park et al., 1996; Park and Gutchess, 2002). With respect to culture, a large number of studies have also demonstrated considerable cross-cultural differences in cognition (Nisbett et al., 2001). Westerners (e.g., North Americans and Western Europeans) tend to be analytic in their reasoning – focusing on a salient object, separating it from the context, and basing their reasoning on logical rules, whereas East Asians (e.g., Koreans, Japanese, and Chinese) tend to be holistic – broadly attending to the entire context and basing their reasoning on experiential knowledge (e.g., intuition). Although aging and cultural influences on cognition have both been well-documented, there has been surprisingly little investigation of their interactions (see Park and Gutchess, 2002 or Chua et al., 2006 for notable exceptions). Therefore, the present research attempted to examine how age and culture combine to jointly influence our cognition.

### Age, Culture, and Cognition

So how might age and culture come together to determine one’s cognition? Park et al. (1999) suggest that in cases where age and culture interact, the form of the interaction will depend on the nature of a task. First, if performance on a culturally sensitive task relies primarily on cultural knowledge (as opposed to cognitive resources), cultural effects on cognition may be well-maintained through late adulthood, since acquired knowledge (e.g., vocabulary) is less likely to show typical age-related declines (Park et al., 1996). Consistent with this notion, recent research has found that both younger and old participants in Japan and the US showed comparably sized cultural differences on cognitive tasks which are sensitive to cultural experiences but not necessarily demanding of cognitive resources (Kitayama et al., 2013, unpublished). For example, they examined participants’ preferences for analytic vs. holistic reasoning (e.g., taxonomic vs. thematic categorization). That is, the tasks used in their research measured cognitive styles (i.e., preferences shaped by cultural knowledge) rather than cognitive abilities and, in these tasks, comparable cultural differences were maintained across the lifespan. Notably, they found that one’s responses in these tasks were not associated with basic cognitive functions such as processing speed. In this sense, these tasks can be said to be relatively insensitive to cognitive resources.

However, Park et al. (1999) also suggest that the interaction between age and culture may take a different form. Specifically, they predicted that cultural differences would be minimized with age due to the strong and universal effects of age-related decline on the task (“biological leveling”), if a task where cultural differences are observed in young is demanding of cognitive resources such as working memory and processing speed. In line with this proposition, Hedden et al. (2002) examined both speed and working memory and reported the evidence of biological leveling –that is, culture effects decreased with age. More specifically, Chinese young adults outperformed American young adults in numerically based tasks, namely the digit comparison as a processing speed task and the digit span as a working memory task. The differences were due to the fact that Chinese syllables for numbers impose a lower processing load than American syllables for numbers (Cheung and Kemper, 1993). More importantly, however, these cultural differences were not observed between Chinese and American older adults (over 60).

It is noteworthy that the nature of a given task is largely determined by the way it is set up. For example, an attribution task can be made up mainly as a cognitive style measure (i.e., low sensitivity to cognitive resources) by investigating one’s relative preference between dispositional and situational explanations without any clear indication that one is more accurate than the other. Or alternatively, an attribution task can be presented in a resource-dependent way (i.e., high sensitivity to cognitive resources) by measuring one’s ability to overcome cultural bias toward dispositional or situational explanations when one is clearly more accurate than the other. In other words, cultural differences in attribution typically found among young adults may or may not be observed among older adults depending on how a given attribution task is set up.

To sum up, previous literature has identified two different forms of the age × culture interaction: (1) no interaction with age (i.e., cultural differences in cognition are held across the lifespan) occurs when the focus is on cognitive styles (e.g., Kitayama et al., 2012, unpublished) and (2) a convergent pattern (i.e., cultural differences in cognition decrease as a function of age) occurs when a given task is sensitive to cognitive resources and age-related declines dilute cultural differences among older adults (e.g., Hedden et al., 2002). Building on the literature, we propose that the interaction between age and culture may take yet another form. Specifically, we predicted that cultural differences in cognition could diverge with aging (i.e., cultural differences in cognition could be larger among older adults than among younger adults). We further argue that this type of interaction between age and culture should occur when a culturally sensitive task makes relatively low demands on cognition. In this case, young adults from the two cultures might have sufficient cognitive resources to overcome biases that their cultural mode of reasoning may produce, but older adults would not, resulting in the interaction.

For example, if East Asian/holistic reasoning leads to better performance in a given task than American/analytic reasoning, American participants may face cultural disadvantages in performing the task. However, if the task is easy, young Americans would be able to overcome any cultural disadvantage that the task may impose, since they have sufficient resources to mitigate such disadvantage. In that case, cultural differences between young Americans and East Asians would be small at best. In contrast, however, older Americans may not be able to overcome cultural disadvantages due to the decline in cognitive resources, which would lead to greater cultural differences between older Americans and East Asians. Note that cultural psychologists have thus far been more interested in cognitive style than cognitive performance and hence, in most tasks they have designed, cognitive load or task difficulty is not directly relevant to the magnitude of cultural differences. Following this logic, the present research investigated whether the predicted pattern of the age × culture interaction (i.e., divergence with aging between cultures) would occur when a given task is sensitive to both cultural experiences/cognitive style and cognitive resources/cognitive performance but at the same time, when the cognitive load for the task is low for younger adults.

Two separate lines of research provide initial support for this predicted form of the age × culture interaction. The first line of work that is relevant to the present analysis comes from a literature of cultural differences in the correspondence bias (i.e., biased toward dispositional attribution in social perception). This literature examines the moderation effect of cognitive load on the magnitude of cultural differences. For example, the correspondence bias was much more pronounced among Westerners than East Asians (Choi et al., 1999) and moreover, Knowles et al. (2001) found that such cultural differences in correspondence bias was magnified by cognitive load since it is cognitively demanding for Americans to take into account situational constraints. Similarly, old Americans showed the largest correspondence bias (Blanchard-Fields et al., 2007). These finding clearly demonstrates that cognitive load interacts with cultural style of reasoning and that age-related declines can be a critical factor in cultural differences in cognition. Secondly, another relevant line of findings was reported in the literature on cognitive aging (Gutchess et al., 2006; Yang et al., 2013). For example, Gutchess et al. (2006) showed that East Asians organized knowledge based on taxonomic categories less than did Americans and further, this tendency was particularly evident among older East Asians presumably because of age-related declines in cognitive resources. Their findings are highly consistent with ours. Building on their findings, the present work directly measured cognitive function and investigated whether cognitive function would interact with culture in a way that we predicted.

### Present Research

In order to investigate the hypothesized form of the age × culture interaction, we studied the *typicality* effect in category judgments – a reasoning task which shows robust cultural differences and also relies on cognitive resources. The typicality effect occurs when people project unknown features from a superordinate category (e.g., bird) to subordinate categories (e.g., eagle or penguin) more easily for typical members as opposed to atypical members (Sloman, 1993). For example, given that all birds have ulnar arteries, people thought it was more likely that all eagles have ulnar arteries than that all penguins have ulnar arteries because an eagle is more typical than a penguin as a member of the bird category. Importantly, the typicality effect is shown to be sensitive to cultural influences (Norenzayan et al., 2002). They found that East Asian culture promotes intuitive reasoning and consequently, East Asians are more susceptible to the typicality effect than Americans. The effect can be also resource-sensitive, as one is required to inhibit an intuitive judgment (based on typicality) and apply logical rules. Hence, we hypothesized that older adults might be more disrupted by the typicality effect than younger adults, since older adults have difficulties in inhibition across various domains (Hasher and Zacks, 1988; Spieler et al., 1996). Taken together, the typicality effect can be sensitive to both aging and cultural influences and provides a rare opportunity to test the age × culture interaction. Therefore, in the present work, we recruited both young and old participants in the US and Singapore and had them make category judgments (i.e., whether a target belongs to the category of living organisms) for either typical targets (living and animate) or atypical targets (living and yet inanimate). Moreover, it is important to note that this type of category judgments is a simple semantic judgment and its cognitive loads are likely to be low for younger adults. Thus, we expected that both age-related cognitive decline and cultural modes of reasoning might reveal the predicted pattern of the age × culture interaction.

In addition, we expected that cultural difference in category judgment would be closely linked to cultural values, since we reasoned that those who endorsed East Asian cultural values would also show East Asian reasoning (i.e., being intuitive). Furthermore, we predicted that the association between cultural values and reasoning would be moderated by cognitive function. That is, a person who highly endorses values in East Asian culture would be more likely to adopt intuitive reasoning and thus, to be particularly vulnerable for the typicality effect. However, such links between cultural values and reasoning would be attenuated if he or she had enough cognitive resources to overcome the bias resulting from the intuitive reasoning. In order to test this moderated mediation model, we also measured participants’ cognitive function and value endorsement.

In sum, the present research investigated the interaction between aging and culture in the domain of categorization. Given that aging and culture are, respectively, neurobiological and experiential processes, the present investigation could demonstrate the dynamic interplay between neurobiological decline (aging) and experiential factors (culture).

## Materials and Methods

### Participants

One hundred and five Americans (US) and 98 Chinese Singaporeans (SG) participated in the study. Younger US (*N* = 51) and SG (*N* = 49) participants were college students. Older participants in both cultures (US: *N* = 54 and SG: *N* = 49) were recruited via community organizations and advertisements. All participants were screened for psychological and physical health. Specifically, volunteers were excluded if there was (1) evidence of psychiatric illness within the past 2 years, including substance abuse, (2) a history of recreational drug use in the previous 6 months, (3) less than 10 years of education, (4) less than 20/30 vision after correction, and (5) a history of CNS disease or brain injury. Also, those who scored lower than 26 on the Mini Mental State Exam (MMSE; Folstein et al., 1975) were excluded. Therefore, it can be said that our participants were physically and psychologically healthy.

### Procedures

#### Category Judgment

Participants were presented with 64 English-word stimuli^1^ and instructed to indicate whether or not each stimulus was a living organism by pressing a designated key. Each word was displayed for 3 s followed by a 500 ms fixation. Half of words were non-living objects, whereas the others were living objects. Among the 32 living objects, six atypical targets were embedded and these items were the focus of the present study (see the **Appendix** for details). The items were atypical in that they were living but inanimate organisms (e.g., seaweed) and were contrasted with typical items that were living and animate (e.g., bear). Given that East Asians are culturally encouraged to be intuitive, we reasoned that the atypical trials would pose a bigger problem for Singaporeans than to Americans. However, young Singaporeans would be able to successfully address such cultural disadvantage since the task would be fairly easy for them and thus, they would have enough cognitive resources to overcome it. Therefore, we predicted the age × culture interaction such that cultural differences would be minimal among young participants but evident among older participants. To ensure that cognitive loads for the task were low, we did not include any ambiguous items.

#### Cognitive Function

In order to characterize age and cultural differences in cognitive function, we included two processing-speed tasks (i.e., Pattern and Dot Matching tasks), a reasoning task (the Cattell Culture-Fair task; Cattell, 1949), and two memory tasks (i.e., Spatial Span and Word) (see Chee et al., 2010 for details). To create a summary index for cognitive function, we ran a principle component analysis (PCA) on the measures of speed, working memory, and reasoning that comprised the cognitive battery. The PCA yielded two components. Since the first component accounted for 53% of variances and all the measures were well-loaded on it (0.63 < all loadings < 0.80), the factor scores of the first component was used as a proxy for cognitive ability (note that the second component accounted for only 15% of the variance).

#### Cultural Values

To characterize cultural differences in values, we administered the Schwartz Value Survey (SVS; Schwartz, 1992). Since we reasoned that those who endorsed East Asian cultural values would also show East Asian reasoning (i.e., being intuitive), we focused on one higher-order value type that we believe to be highly endorsed in East Asia, namely conservation (a set of values justifying the belief that people are truly embedded in their groups; Schwartz, 1999). Consistent with this reasoning, previous study showed that conservation was more endorsed by East Asian cultures such as China and Korea than by English-speaking cultures such as American and Canada (Schwartz, 1999). Conservation consists of three value types, Tradition, Conformity, and Security (Schwartz, 1994). In the SVS, there are 14 items measuring these 3 value types, Tradition (5 item such as Humble and Moderate), Conformity (4 items such as Honoring Parents and Elders and Obedient), and Security (5 items such as Social Order and Sense of Belonging). Participants reported personal importance of each item on a 9-point scale (-1: Opposite of what I value to 7: Extremely Important). In our data, the reliability of conservation was acceptable in both cultures (US: α = 0.81 and SG*:*α = 0.86). Thus, the index of conservation was created by averaging them.

## Results

### Sample Characteristics

#### Demographic Information

Demographic variables such as age and gender did not vary significantly across two cultures except for education years. A 2 (Age: Young vs. Old) × 2 (Culture: SG vs. US) ANOVA on education years revealed a main effect of culture (US > SG), *F*(1,199) = 21.10, *p* < 0.001, $\eta_{p}^{2}$ = 0.10. Moreover, this culture effect was qualified by a significant Age × Culture interaction, *F*(1,199) = 16.55, *p* < 0.001, $\eta_{p}^{2}$ = 0.08, such that old Americans (*M* = 15.46) were more educated than old Singaporeans (*M* = 12.74), *t*(101) = 5.26, *p* < 0.001, *d* = 1.05, while there was no difference between young Americans (*M* = 14.52) and young Singaporeans (14.33). Also, the main effect of age was not significant. Taken together, participants from each culture were comparable in terms of demographic variables except that older American participants were better educated than old Singaporean participants (see **Table 1** for detailed information). The difference in education years may be critical because education could influence their performance in the category judgment task and thus, education years were included in the main analyses (reported below) as a covariate. This issue is further addressed in the discussion section.

**Table 1:** Demographic information.

	US		Singapore	
		
	Young (51)	Old (54)	Young (49)	Old (49)
Gender	M: 26 F: 25	M: 25 F: 29	M: 25 F: 24	M:24 F: 25
Mean age	22.03	66.61	24.22	65.96
Age range	20–29	61–78	20–30	61–76
MMSE	29.08 (1.01)	28.28 (1.18)	29.38 (0.92)	28.30 (1.16)
Education years	14.52 (1.83)	15.46 (2.58)	14.33 (1.52)	12.74 (2.66)


#### Cognitive Function

A 2 (age) × 2 (culture) ANCOVA on the index with education years as a covariate revealed that first, there was no cultural difference in cognitive function, *F* < 1; second, older participants scored lower than younger participants, *F*(1,198) = 303.64, *p* < 0.001, $\eta_{p}^{2}$ = 0.61 and finally, the age effect was not qualified by culture, *F* < 1, US: Ms = 0.79 (Young) vs. -0.68 (Old), *t*(103) = 11.97, *p* < 0.001, *d* = 2.36, and SG: Ms = 0.80 (Young) vs. -0.82 (Old), *t*(96) = 12.84, *p* < 0.001, *d* = 2.62. In other words, in terms of cognitive function, there was no cultural difference and age-related cognitive declines were comparable across two cultural groups. Also, note that educations years did not have significant impact on cognitive function, *F*(1,198) = 1.09, *p* = 0.299, $\eta_{p}^{2}$ = 0.005.

#### Cultural Values

A 2 (age) × 2 (culture) ACNOVA on the index of conservation with education years as a covariate found a main effect of age (Old > Young), *F*(1,198) = 59.84, *p* < 0.001, $\eta_{p}^{2}$ = 0.23, and a main effect of culture, *F*(1, 198) = 62.53, *p* < 0.001, $\eta_{p}^{2}$ = 0.24. Also, the interaction between age and culture was not significant, *F* < 1. Thus, confirming our prediction, Singaporean were higher on conservation than Americans both for younger participants: SG = 19.48 vs. US = 15.54, *t*(98) = 6.00, *p* < 0.001, *d* = 1.21 and for older participants: SG = 23.33 vs. US = 19.17, *t*(101) = 5.96, *p* < 0.001, *d* = 1.19. We also note that cultural values did not significantly vary as a function of education years, *F* < 1.

### Category Judgment

#### Accuracy

As the first step^2^ in the analysis of the category judgment data, we conducted a 2 (Age: Young vs. Old) × 2 (Culture: SG vs. US) × 2 (Type: Animate vs. Inanimate) mixed ANCOVA on the overall accuracy with type as a within-subject factor and education years as a covariate. The ANCOVA did not find any significant effect. That is, the overall accuracy did not vary as a function of trial type, age and culture. Rather, there was a high rate of accuracy, ranging from 0.96 to 0.99 among the four groups. In other words, a ceiling effect was observed, which confirms our expectation that the category judgment task would be easy enough for all the participants. More importantly, this result allowed us to test our critical prediction, whether cultural differences would be significant for older adults yet negligible for younger participants when a given task is easy.

#### Reaction Time

We predicted that Singaporeans would show larger typicality effects (slower RT for inanimate than for animate organisms) than Americans and yet, these cultural differences would be less (or not) evident for younger participant than for older participants. In order to test the prediction, we conducted a 2 (Age: Young vs. Old) × 2 (Culture: SG vs. US) × 2 (Type: Animate vs. Inanimate) mixed ANCOVA on the reaction times (RTs) data with type as a within-subject factor and education years as a covariate. The critical test of our prediction is whether the three-way interaction would turn out to be significant in this mixed ANCOVA. Confirming our prediction, the results showed the significant three-way interaction, *F*(1,198) = 4.93, *p* = 0.028, $\eta_{p}^{2}$ = 0.024. We also note that education years again did not show any significant effect^3^.

**FIGURE 1:**
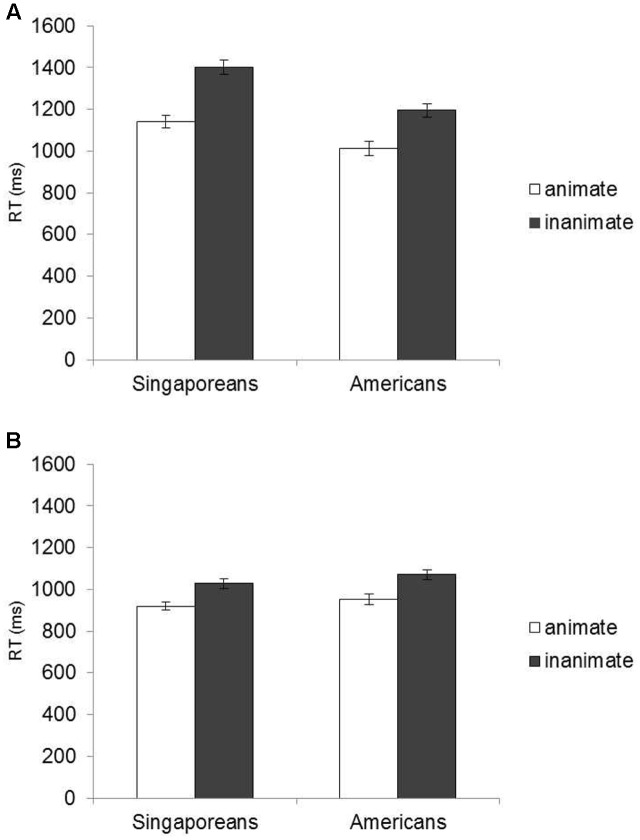
**Mean RTs in the category judgment task by culture:** **(A)** old and **(B)** young participants.

In order to further explore the three-way interaction effect, we conducted a 2 (Culture: SG vs. US) × 2 (Type: Animate vs. Inanimate) mixed ACNOVA on RTs for each age group. Among older participants, as shown in **Figure 1A**, the ANOVA found a main effect of Culture–older Singaporeans were slower than older Americans, *F*(1, 100) = 14.32, *p* < 0.001, $\eta_{p}^{2}$ = 0.13. More importantly, the Culture × Type interaction was significant, *F*(1, 100) = 9.11, *p* = 0.003, $\eta_{p}^{2}$ = 0.084. It occurred because the difference between Singaporeans and Americans were significantly larger for inanimate organisms (RTs: 1400.73 vs. 1195.73 ms, *t*(101) = 4.37, *p* < 0.001, *d* = 0.86) than for animate organisms (RTs: 1138.91 vs. 1011.01 ms, *t*(101) = 3.65, *p* < 0.001, *d* = 0.73). In other words, the predicted cultural differences in the typicality effect were confirmed. In contrast, among younger participants, a 2 (Culture) × 2 (Type) ANCOVA only found a main effect of Type (RTs: inanimate > animate organisms) *F*(1,97) = 7.60, *p* = 0.007, $\eta_{p}^{2}$ = 0.073. That is, for both young Singaporeans and Americans showed the typicality effect to a similar degree, RTs (ms): 1027.95 vs. 919.93, *t*(48) = 6.19, *p* < 0.001 for Singaporeans and 1071.27 vs. 952.79, *t*(50) = 5.64, *p* < 0.001^4^.

Another way to look at the three-way interaction is conducting a 2 (Age) × 2 (Type) ANCOVA within each culture. These ANCOVAs revealed a significant Age × Type interaction effect for both Singaporeans, *F*(1,95) = 22.41, *p* < 0.001, $\eta_{p}^{2}$ = 0.19, and Americans, *F*(1,103) = 3.75, *p* = 0.056, $\eta_{p}^{2}$ = 0.04. This suggests that the typicality effect was larger for older participants than for younger participants in both cultures. However, as indicated in the significant three-way interaction effect of the overall ANCOVA, the age-related difference in the typicality effect was more pronounced among Singaporeans than among Americans.

Taken together, a series of ANCOVA analyses on RTs showed the predicted pattern of the Age × Culture interaction. Consistent with our prediction, cultural differences were negligible among younger participants but were revealed in older participants. We argued that this occurred because (1) our task was a performance measure, favoring Americans and yet, (2) it was a simple semantic judgment and hence, its cognitive loads were low, especially for younger participants (for example, the overall accuracy ranged from 0.96 to 0.99 among the four groups). We believe that this allowed young Singaporeans to overcome cultural disadvantages.

### Cognitive Function and Cultural Values

**FIGURE 2:**
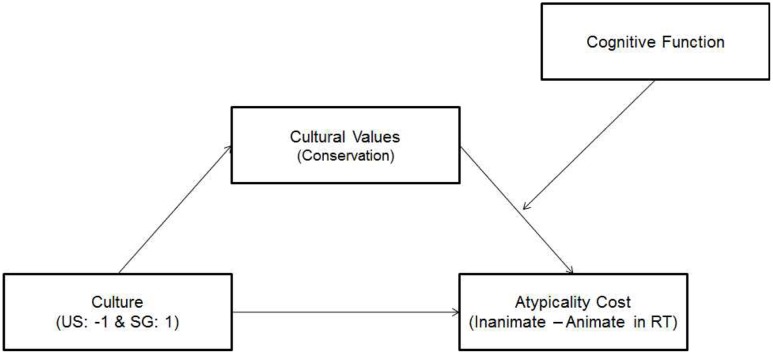
**Moderated mediation.** One’s endorsement of culture interacts with cognitive function to predict his or her performance in the category judgment task.

In order to investigate a possible mechanism underlying the cultural differences in the previous section, we conducted a moderated mediation analysis using the Process macro (Hayes, 2013). First, we reasoned that the typicality effect was stronger for Singaporeans than for Americans because Singaporean endorsed East Asian culture and hence, intuitive reasoning. In other words, cultural differences in the typicality effect would be mediated by participants’ endorsement of East Asian culture, as measured by cultural values. However, this mediation effect would be moderated by cognitive function as those with enough cognitive resources (e.g., young Singaporeans) should overcome the disadvantage of cultural reasoning (i.e., intuitive reasoning). This implies a moderated mediation model depicted in **Figure 2** (Model 14). To test this model, the cost of atypicality (the relative cost of inanimate organisms in RTs) was calculated by subtracting RTs for animate organisms from RTs for inanimate organisms. Then, this atypicality cost was used as a dependent variable in the moderated mediation model. In the model, culture was used as the independent variable (Singaporeans = -1; Americans = 1), cultural values (i.e., conservation) as the mediator, and cognitive function as the moderator of the cultural value (i.e., mediator) to the atypicality cost (i.e., DV). Education years were also included as a covariate. Since it is critical to our prediction to compare participants with good cognitive function and those with poor cognitive function, we estimated the conditional indirect effect at one standard deviation above and below the mean of cognitive function.

The analyses reported in **Tables 2**,**3** supported our hypothesis. First, as shown in **Table 2**, culture significantly predicted one’s endorsement of conservation (the mediator model) and also, one’s endorsement of conservation interacted with cognitive function to predict the typicality cost (the DV model). More importantly, the conditional indirect effects in **Table 3** showed that the indirect effect of culture on the atypicality cost through one’s endorsement of conservation was significant for those with poor cognitive function (i.e., -1 SD), as indicated in the CI that did not include zero. In contrast, the CI for the indirect effect included zero and hence, was not significant for those with good cognitive function (i.e., +1 SD). As inferred in this patter, the 95% confidence interval of the index of moderated mediation (Hayes, 2015) did not include zero suggesting significant moderated mediation, Index = -9.59, SE = 5.37, 95% CI [-22.03, -0.34]. In other words, Singaporeans with poor cognitive function suffered more from the typicality effects than Americans because of their endorsement of East Asian culture whereas Singaporeans with good cognitive function could overcome disadvantage of East Asian cultural reasoning. This is consistent with our interpretation of the age × culture interaction on the typicality (i.e., only older participants showed cultural differences because of their relatively poor cognitive function).

**Table 2:** Regression coefficients in the moderated mediation model.

Model	*B*	*SE*	β	*t*	*p*
Mediator model		**(Mediator = Conservation)**			
Constant	0.29	0.40		0.72	0.472
Culture	0.46	0.06	0.46	7.33	0.000
Education years	-0.019	0.03		-0.69	0.492
DV model		**(DV = Atypicality cost)**			
Constant	102.44	69.48		1.47	0.142
Culture	7.01	12.92		0.54	0.588
Conservation	25.89	13.91		1.86	0.064
Cdognitive function	-43.75	12.43		-3.52	0.001
Conservation × Cognitive function	-21.58	10.86		-1.99	0.048
Education years	3.95	4.76		0.82	0.408


*Note that Conservation and Cognitive function were standardized.*

**Table 3:** Conditional indirect effect at one standard deviation below and above the mean of cognitive function.

Cognitive function	Effect	SE	95% CI
-1 SD	21.11	8.78	[6.52, 41.45]
+1 SD	1.92	7.27	[-12.30, 16.79]


### Alternative Explanations

**FIGURE 3:**
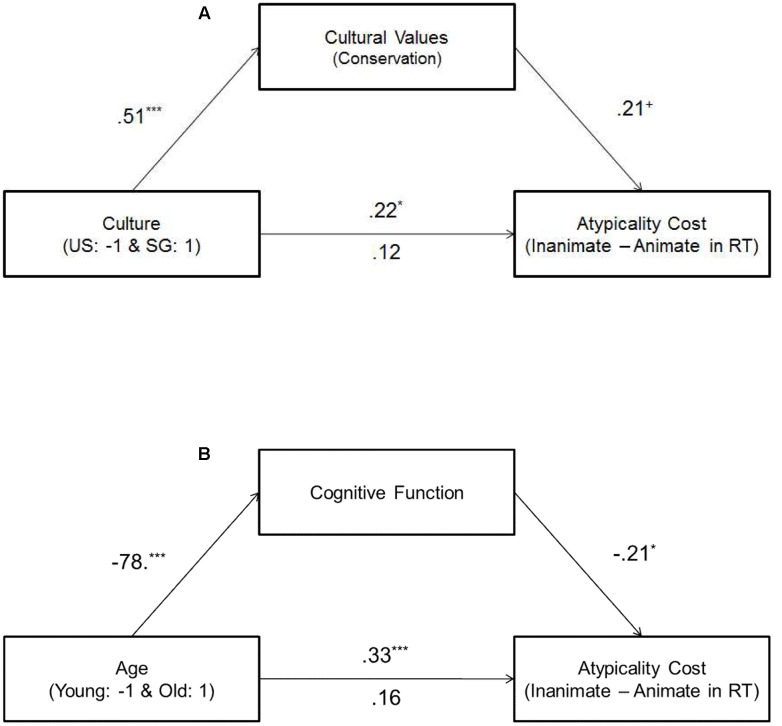
**Mediation analyses:** **(A)** the mediating effect of cultural values on the cultural difference and **(B)** the mediating effect of cognitive function on the age difference, ^∗∗∗^*p* < 0.001, ^∗^*p* ≤ 0.05 and ^+^*p* = 0.07.

Next, we addressed a couple of alternative explanations for our findings. First, one may suspect that differences in cognitive function are associated with cultural differences in the typicality effects we found among older participants. However, among older participants, cognitive function did not vary across cultures, β = -11, *p* > 0.20 and also, cultural differences remained significant even when controlling for cognitive function, β = 0.21, *p* = 0.04. Instead, our data suggest that the cultural difference in category judgment among older adults was mediated by cultural values. Specifically, culture significantly predicted cultural values (conservation), β = 0.51, *p* < 0.001 (SG → more endorsement of East Asian values), and cultural values, in turn, predicted the atypicality cost, even after controlling for culture, β = 0.21, *p* = 0.07. Furthermore, when controlling for cultural values, the culture effect on the cost decreased from β = 0.22, *p* = 0.02 to β = 0.12, *ns.* Moreover, the Sobel test showed that the mediating effect of cultural values was marginally significant, *Z* = 1.77, *p* = 0.08 (**Figure 3A**).

Second, some may argue that age differences in the typicality effect are closely linked to conservation (i.e., East Asian values) as opposed to cognitive function because conservation would be more endorsed by older participants than by younger participants in general. Since there was no Age × Culture interaction on cognitive function, we collapsed the data across cultures and tested this idea. Contrary to the argument that the age differences in the typicality effect might be driven by conservation, the age effect on the atypicality cost remained significant even after controlling for conservation, β = 0.25, *p* = 0.001, although conservation was associated with the atypicality cost, β = 0.28, *p* < 0.001. Instead, cognitive function significantly predicted the atypicality cost in RT after controlling for age, β = -0.21, *p* = 0.045 (less cognitive ability → more cost). Moreover, as predicted, there was no effect of age on the atypicality cost after controlling for cognitive function, β = 0.16, *ns*. Finally, the Sobel test showed that the mediating effect of cognitive function was significant, *Z* = 1.95, *p* = 0.05 (**Figure 3B**). Thus, as predicted, the age difference in category judgment was mediated by cognitive function.

Taken together, a series of mediation analyses shows that aging and culture both play an important role in cognition and yet, different processes underlie the effects (cognitive function for the age effect and cultural values for the culture effect). More importantly, as the moderated mediation analysis suggests, cognitive function and cultural values interacted in the predicted way, such that a participant’s endorsement of culture mattered only for those with poor cognitive function when dealing with a relatively easy task. However, we note that cross-sectional mediation analyses should be interpreted with greater caution than longitudinal data (Spencer et al., 2005; Bullock et al., 2010), especially with respect to aging (Lindenberger et al., 2011). Despite this limitation, the present data suggested that age and culture can interact with each other to jointly influence one’s reasoning.

## Discussion

To our knowledge, the present research is a rare examination of the age × culture interaction in cognition, demonstrating that a reasoning task is jointly affected both by cultural knowledge and cognitive resources. We found that older adults performed worse than did younger adults on an atypical judgment task that require inhibiting an intuitive categorization judgment. Although both young Singaporeans and young Americans performed similarly on this task, we observed culture effects in older adults. Older, but not younger, Singaporeans showed the typicality effect more than did their counterparts in the US. Moreover, we also showed that cultural values and cognitive function interacted with each other to influence cognition such that cultural values made a difference for those without sufficient cognitive resources, but not for those with sufficient cognitive resources to deal with relative cultural disadvantages.

The first implication of the current findings is that cultural biases not present in young adults may be revealed with age. The task used in the present research was quite easy (mean RT for even the old Singaporeans was less than 1.5 s and their accuracy was over 97%). We argue that younger people are able to overcome mild disadvantages that a culturally incompatible task imposed, as long as cognitive loads for the task are low. In line with this reasoning, present work found that the cultural difference in the task was negligible among younger participants, whereas it became evident among older participants. As such, present work suggests that various factors (e.g., age, cognitive function, cultural knowledge, and task difficulty) should be taken into account to fully understand the interplay between age and culture. Systematic investigation of these issues would be a worthy endeavor for future work.

Another implication of the current work is suggesting that different processes may be responsible for aging and for cultural effects on cognition. A series of regression analyses showed that aging effects are closely linked to age related declines in cognitive function whereas cultural influences are highly associated with one’s endorsement of their culture. Firstly, across the two cultures, the age differences in the atypicality cost disappeared when controlling for cognitive function, suggesting that age-related declines in cognitive function may mediate the corresponding age-differences in the atypicality cost. Second, our data also showed that cultural differences between older Singaporean and American participants disappeared when controlling for cultural values, suggesting that cultural values may mediate cultural differences in the atypical cost. More importantly, we provided empirical evidence showing that cultural values and cognitive function jointly influence our cognition. Specifically, the moderated mediation showed that East Asian values (i.e., conservation) mediated a cost of East Asian reasoning (i.e., atypicality cost) only when one did not have enough cognitive resources to overcome cultural disadvantages. Then, this finding shows how cognitive function and culture interact with each other to determine our cognitive processes.

Before closing, several cautionary notes seem warranted. First, there was an age × culture interaction effect on education years that closely resembled the key interaction effect we found in the atypicality effect. Namely, old American participants were more educated than old Singaporean participants. This may be problematic as education is closely associated with cognitive performance. However, our old Singaporeans received more education, compared to old Singaporeans in other similar studies (e.g., Chee et al., 2009). Also, in spite of less education, old Singaporeans did not significantly differ from old Americans in terms of basic cognitive functions such as processing speed or working memory. Moreover, the key interaction between age and culture remained significant after controlling for education years. Similarly, it may be problematic to use English materials for Singaporeans although both young and old participants in the present research were fluent in English. Thus, these issues regarding education years and native language should be further addressed in future work. Second, although atypical trials constitute the critical condition of the present work, its number may be not enough to test the key interaction. However, variations among atypical trials were comparable to those among typical trials that had enough number of trials, as indicated in the error bars of **Figure 1**. This suggests that the small number of atypical trials might not be a serious issue in the present research. Third, the present work focused on age-related cognitive decline and yet, wise reasoning has been show to improve into old age presumably because of life experiences (Grossmann et al., 2010). Thus, it would be a worthy endeavor for future research to investigate how culture may interact with age-related improvement in reasoning, as shown in a recent study (Grossmann et al., 2012). Finally, the present research is cross-sectional and thus, it is difficult to rule out other potential generations effects. Thus, this issue should be clarified in future work with a longitudinal design.

To conclude, the results clearly demonstrated the joint effect of biological processing (aging) and sustained experiences (culture) on cognition. As Park and Gutchess (2002) proposed, the Age × Culture interaction can provide a unique opportunity to examine how biological and experiential factors may come together to act on our reasoning, since aging influences in cognition rely mostly on biological factors and cultural influences rely largely on experiential factors. Therefore, the age × culture interaction is an important domain for systematic investigation and an effort to understand the impact of neurobiology and situated context on cognition. We hope that the present work adds to the emerging literature on such investigations (Kitayama et al., 2013, unpublished).

## Ethics Statement

This study was carried out in accordance with the recommendations of the Institutional Review Board at the University of Illinois at Urbana-Champaign with written informed consent from all subjects. All subjects gave written informed consent in accordance with the Declaration of Helsinki. The protocol was approved by the same IRB.

## Author Contributions

All authors developed the study concept and contributed to the study design. JN performed the data analysis and all authors discussed the results. JN drafted the manuscript and the other authors provided comments and revised the manuscript.

## Conflict of Interest Statement

The authors declare that the research was conducted in the absence of any commercial or financial relationships that could be construed as a potential conflict of interest.

## Footnotes

1 It may be argued that using English is problematic because of potential differences in English fluency between young and old Singaporeans. However, English is the official language in Singapore and thus, both young and old Singaporeans were fluent in English. For example, old Singaporeans performed as well as old Americans in our cognitive function tasks although they were tested with English. Further, we predicted that old Singaporeans would be slower for the atypical trials than for the typical trials and yet, there is no reason to believe that English fluency would be particularly problematic for the atypical trials. All in all, English fluency is not an issue in interpreting the results in the present research.

2 Mean RTs were used but median RTs also produced the same pattern of results. Also note that mean RTs were calculated for correct responses and outliers within each participant (mean ± 2.5 SD) were excluded.

3 In addition to the three-way interaction effect, other effects (main effects of age and culture as well as age × type, age × culture interaction effects) were significant, all *F*s > 4.70, all *p*s < 0.05. The nature of these effects were revealed and discussed in the following analyses.

4 It may seem odd that we did not find any cultural difference among younger participants. In fact, this may be inconsistent with the previous study (e.g., Norenzayan et al., 2002) reporting cultural difference in categorization. However, even in the previous study, cultural difference was found when the task was framed as a similarity judgment, but not when the task was framed as a classification judgment (e.g., Study 2 in Norenzayan et al., 2002; see also Gutchess et al., 2006). Thus, when a categorization task is introduced as a formal test with correct answers (as in our study), it is not uncommon to fail to find cultural difference among younger adults who are taught formal logic (e.g., college students).

## APPENDIX

Words used in the category judgment task.

*Animate living objects*: baby, banker, bear, beaver, blacksmith, camel, clown, cook, crow, doe, dove, duchess, geese, horse, lawyer, miner, mosquito, moth, mule, nun, pup, queen, robber, toad, uncle, walrus

Inanimate living objects: flower, elm, dandelion, plant, seaweed, shrug
